# Metabolic syndrome and depressive symptoms among rural Northeast general population in China

**DOI:** 10.1186/s12889-016-3913-0

**Published:** 2017-01-06

**Authors:** Shasha Yu, Hongmei Yang, Xiaofan Guo, Liqiang Zheng, Yingxian Sun

**Affiliations:** 1 Department of Cardiology, The First Hospital of China Medical University, Shenyang, Liaoning China; 2 Department of Clinical Epidemiology, Shenjing Hospital of China Medical University, Shenyang, Liaoning China

## Abstract

**Background:**

Previous researches aiming to estimate the association between metabolic syndrome and depressive symptoms come out with inconsistent results. Besides, most of them are conducted in the developed areas. There is lack of the data from rural China. The aim of this study is to confirm whether gender difference exists among the relationship between MetS, metabolic components and depressive symptoms in the rural Chinese population.

**Methods:**

A cross-sectional analysis enrolled 11430 subjects’ aged ≥35 from rural Northeast China. Metabolic and anthropometric indicators were measured according to standard methods. Depressive symptoms were defined using the Patient Health Questionnaire-9 (PHQ-9).

**Results:**

The prevalence of depressive symptoms was 6% among rural Northeast general population and the prevalence of MetS and its components were 39.0% for MetS, 42.9% for abdominal obesity, 67.1% for elevated blood pressure, 47.1% for hyperglycemia, 32.1% for hypertriglyceridemia, 29.5% for low HDL-C. Depressive symptoms were associated with triglyceride component (OR = 1.24, 95%CI: 1.05–1.46, *P =* 0.01) but not MetS (OR = 1.11, 95%CI: 0.94–1.30, *P* = 0.23). Moreover, depressive symptoms were associated with triglyceride component (OR = 1.21, 95% CI = 1.00–1.47, *P =* 0.05) in women only. But once adjusted for menopause status, depressive symptoms were no longer statically associated with triglyceride component (OR = 1.20, 95% CI = 0.99–1.46, *P* = 0.07).

**Conclusions:**

Depressive symptoms were associated with triglyceride component but not MetS in rural Chinese population. Routine lipid screening should be recommended among rural depressed residents especially among female.

## Background

Depression represents a major international public health problem. Previous study confirmed that depression contributed significantly to suicidal ideation and was closely associated with all-cause mortality and adverse health outcomes in both developed and developing countries [1]. Two national surveys of mental disorders, conducted in 1982 and 1993, revealed very low, but increasing, lifetime prevalence (0.46 and 0.83%) of affecting disorders in China [2, 3]. Study conducted in Hong Kong claimed that the prevalence of depressive symptom was 17% in those with one or more chronic conditions, and was more prevalent in women than in men (19.7% vs. 13.9%) [4]. In addition to some conventional associations between depressive symptoms and older age, female gender, low education, cognitive impairment, living alone and history of chronic diseases [2, 3], resent studies claimed that depressive symptoms might be associated with metabolic syndrome (MetS) [5, 6]. However, there were other studies did not find any positive association between MetS and psychological distress [7, 8]. However, most of those studies were carried in developed areas or countries and there were lack of data about rural residents in China. Furthermore, it remained controversial whether gender difference played any role in the relationship between MetS and depressive symptoms. There was cross-sectional study demonstrated that depressive symptoms were associated with MetS, irrespective of gender [9]. But Sekita A and colleagues reported that the association between MetS and depressive symptoms existed only in a general population of Japanese men but not women which was similar to other studies [5, 10]. On the contrary, Kinder LS and colleagues carried a study in the United States and found that women with depressive symptoms were more likely to have MetS, but this was not found among men with history of depression [11].

In the present study, we aimed to confirm the possible relationship between MetS and depressive symptoms in residents from rural Northeast China and aimed to clarify whether gender difference affected the relationship between MetS and depressive symptoms in rural Chinese.

## Methods

### Study population

Liaoning Province is located in Northeast China. From January 2012 to August 2013, a representative sample of participants aged ≥ 35 years was selected to characterize the prevalence, incidence and natural history of cardiovascular risk factors in rural areas of Liaoning Province. The study adopted a multi-stage, stratified, random-cluster sampling scheme. In the first stage, three counties (Dawa, Zhangwu and Liaoyang County) were selected from the eastern, southern and northern regions of Liaoning province. In the second stage, one town was randomly selected from each county (for a total of three towns). In the third stage, 8–10 rural villages from each town were randomly selected (for a total of 26 rural villages). Participants who were pregnant or had malignant tumors or mental disorders were excluded from the study. All the eligible permanent residents aged ≥ 35 years from each village were invited to attend the study (a total of 14,016 participants). Of those, 11,956 participants agreed and completed the study to give a response rate of 85.3%. The study was approved by the Ethics Committee of China Medical University (Shenyang, China, AF-SDP-07-1, 0–01). All procedures were performed in accordance with ethical standards. Written consent was obtained from all participants after they had been informed of the objectives, benefits, medical items and confidentiality agreement regarding their personal information. For participants who were illiterate, we obtained written informed consent from their proxies. In this report, we focused on the hypertensive residents lived in the rural Northeast China, making a final sample size of 11430 (5279 men and 6151 women).

### Data collection and measurements

Data were collected during a single visit to the clinic by cardiologists and trained nurses using a standard questionnaire in a face-to-face interview. Before the survey was performed, we invited all eligible investigators to attend an organized training session. The training included the purpose of this study, how to administer the questionnaire, the standard method of measurement, the importance of standardization and the study procedures. A strict test was administered after this training, and only those who scored perfectly on the test were accepted as investigators in this study. During data collection, our inspectors had further instructions and support.

Data on demographic characteristics, lifestyle risk factors, dietary habits, family income, history of cardiovascular disease, evaluation of psychological status were obtained by interview with a standardized questionnaire. There was a central steering committee with a subcommittee for quality control. Educational level was divided into primary school or below, middle school and high school or above. Family income was classified as ≤5 000, 5 000–20 000 and >20 000 CNY/year. Self-reported sleep duration (including nocturnal and nap duration) was obtained from the questionnaire. The responses were categorized into four groups: ≤7, 7–8, 8–9, and >9 h/d.

According to American Heart Association protocol, blood pressure was measured three times at 2-min intervals after at least 5 min of rest using a standardized automatic electronic sphygmomanometer (HEM-907; Omron Healthcare, Kyoto, Japan), which had already been validated according to the British Hypertension Society protocol [12]. The participants were advised to avoid caffeinated beverages and exercise for at least 30 min before the measurement. During the measurement, the participants were seated with the arm supported at the level of the heart. The mean of three BP measures was calculated and used in all analyses.

Weight and height were measured to the nearest 0.1 kg and 0.1 cm, respectively, with the participants wearing light-weight clothing and without shoes. Waist circumference (WC) was measured at the umbilicus using a non-elastic tape (to the nearest 0.1 cm), with the participants standing at the end of normal expiration. Body mass index (BMI) was calculated as the weight in kilograms divided by the square root of the height in meters.

Fasting blood samples were collected in the morning after at least 12 h of fasting. Blood samples were obtained from an antecubital vein into Vacutainer tubes containing ethylenediaminetetraacetic acid (EDTA). Fasting plasma glucose (FPG), total cholesterol (TC), low-density lipoprotein cholesterol (LDL-C), high-density lipoprotein cholesterol (HDL-C), triglycerides (TGs) and other routine blood biochemical indexes were analyzed enzymatically on an Olympus AU640 autoanalyzer (Olym-pus, Kobe, Japan). All laboratory equipment was calibrated, and blinded duplicate samples were used for these analyses.

### Definitions

As the definition showed in previous study [13], Participants having three or more of the following criteria were defined as having the metabolic syndrome: 1. Abdominal obesity for Asian: ≥90 cm for men and ≥80 cm for women; 2. Elevated triglycerides (drug treatment for elevated triglycerides is and alternate indicator): ≥ 150 mg/dL (1.7 mmol/L); 3. Reduced high-density lipoprotein (HDL) cholesterol (drug treatment for reduced HDL-C is and alternate indicator) : <40 mg/dL (1.0 mmol/L) in men and <50 mg/dL (1.3 mmol/L) in women; 4. Elevated blood pressure (antihypertensive drug treatment in a patient with a history of hypertension is and alternate indicator)): ≥ 130/85 mmHg; 5. Elevated fasting glucose (drug treatment of elevated glucose is an alternate indicator): ≥ 100 mg/dL (≥5.6 mmol/L).

Depressive symptoms were assessed with PHQ-9, which is widely used in primary health centers for the screening of depression. Each of the nine PHQ depression items corresponds to one of the DSM-IV diagnostic criterion for symptoms for major depressive disorder [14]. The subjects were asked how often, over the past 2 weeks, they had been bothered by each of the depressive symptoms. The response options were “not at all”, “several days”, “more than half the days”, and “nearly every day” and were scored as 0, 1, 2, and 3, respectively. PHQ-9 scores range from 0 to 27 and with scores of ≥5, ≥10, and ≥15, representing mild, moderate, and severe levels of depression severity [15]. Individuals with a PHQ-9 score greater than 10 are considered to be suffering from severe depression symptoms [16].

Physical activity included occupational and leisure-time physical activity. A detailed description of the methods has been presented elsewhere [17]. Occupational and leisure-time physical activity were merged and regrouped into 3 categories: (1) low—subjects who reported light levels of both occupational and leisure-time physical activity, light which indicates very easy physical activity, sitting work, e.g., secretary; (2) moderate—subjects who reported moderate or high levels of either occupational or leisure-time physical activity, moderate for work including standing and walking, e.g. Store assistant; and (3) high—subjects who reported a moderate or high level of both occupational and leisure-time physical activity which indicates work including walking and lifting, or heavy manual labour, e.g. Industrial work, farm work.

### Statistical analysis

Descriptive statistics were calculated for all the variables, including continuous variables (reported as mean values and standard deviations) and categorical variables (reported as numbers and percentages). Differences between different groups were evaluated using Student’s *t*-test, ANOVA, non-parametric test or the *χ*2-test as appropriate. Multivariate logistic regression analyses were used to identify association between MetS and depressive symptoms and each of its components with odds ratios (ORs) and corresponding 95% confidence intervals (CIs) calculated. All the statistical analyses were performed using SPSS version 17.0 software SPSS Inc, Chicago, IL, US and *p* values less than 0.05 were considered statistically significant.

## Results

### Demographic and metabolic characteristics

Depressive symptoms were presented in 685 (6.0%) patients, including 186 (3.5%) men and 499 (8.1%) women. Compared with their non-depressive counterparts, more depressed men and women were currently unmarried and ex-smoker. They did not likely to have moderate and server excises. Besides, a larger percentage of depressive subjects had lower educational status. Men with depressive symptoms were significantly more likely to be ex-drinker at the time of the survey compared with men without depressive symptoms. Additionally, both depressive women and men tended to have lower family income than those without depression. Both depressive women and men were significantly older than those without depressive symptoms counterparts. The detailed baseline demographic characteristics of all subjects were shown in Table 1.

**Table 1:** Demographic characteristics of the population by depressive symptoms and sex

	Total		*P*-value^a^	Men		*P*-value^a^	Women		*P*-value^a^
	Depressed	Not depressed		Depressed	Not depressed		Depressed	Not depressed	
	*N* = 685	*N* = 10745		*N* = 186	*N* = 5093		*N* = 499	*N* = 5652	
Age, mean ± SD, years	57.07 ± 10.27	53.61 ± 10.55	<0.001	57.25 ± 11.16	54.24 ± 10.77	<0.001	56.99 ± 9.94	53.04 ± 10.32	<0.001
Ethnicity (Others^a^)	653 (95.3)	10179 (94.7)	0.277	175 (94.1)	4822 (94.7)	0.408	478 (95.8)	5357 (94.8)	0.192
Marriage									
Currently not married, *n* (%)	99 (14.5)	859 (8.0)	<0.001	30 (16.1)	382 (7.5)	<0.001	69 (13.8)	477 (8.4)	<0.001
Cigarette smoking			<0.001			<0.001			<0.001
Current smoker, *n* (%)	198 (28.9)	3836 (35.7)		94 (50.5)	2917 (57.3)		104 (15.1)	919 (16.3)	
Ex-smoker, *n* (%)	43 (6.3)	436 (4.1)		27 (14.5)	348 (6.8)		16 (3.2)	88 (1.6)	
Non-smoker, *n* (%)	444 (64.8)	6473 (60.2)		65 (34.9)	1828 (35.9)		5024 (81.7)	4645 (82.2)	
Alcohol consumption			<0.001			<0.001			0.506
Current drinker, *n* (%)	74 (10.8)	2501 (23.3)		55 (29.6)	2338 (45.9)		19 (3.8)	163 (2.9)	
Ex-drinker, *n* (%)	24 (3.5)	259 (2.4)		22 (11.8)	236 (4.6)		2 (0.4)	23 (0.4)	
Non-drinker, *n* (%)	587 (85.7)	7985 (74.3)		109 (58.6)	2519 (49.5)		478 (95.8)	5466 (96.7)	
Exercise			<0.001			<0.001			<0.001
Light	322 (47.0)	3075 (28.6)		76 (40.9)	1116 (21.9)		246 (49.3)	1959 (34.7)	
Moderate	338 (49.3)	7052 (65.6)		103 (55.4)	3692 (72.5)		235 (47.1)	3360 (59.4)	
Severe	25 (3.6)	618 (5.8)		7 (3.8)	285 (5.6)		18 (3.6)	333 (5.9)	
Educational status			<0.001			0.02			<0.001
Primary school or below	452 (66.0)	5237 (48.7)		94 (50.5)	2105 (41.3)		358 (71.7)	3132 (55.4)	
Middle school	203 (29.6)	4459 (41.5)		79 (42.5)	2396 (47.0)		124 (24.8)	2063 (36.5)	
High school or above	30 (4.4)	1049 (9.8)		13 (7.0)	592 (11.6)		17 (3.4)	457 (8.1)	
Annual income (CNY/year)			<0.001			<0.001			<0.001
≤5000	173 (25.3)	1249 (11.6)		61 (32.8)	648 (12.7)		112 (22.4)	601 (10.6)	
5000–20000	380 (55.5)	5840 (54.4)		95 (51.1)	2733 (53.7)		285 (57.1)	3107 (55.0)	
>20000	132 (19.3)	3656 (34.0)		30 (16.1)	1712 (33.6)		102 (20.4)	1944 (34.4)	

Abbreviations: *SD* standard deviation. ^a^Two-tailed Student’s *t*-test for normally distributed variables and chi-square test for categorical variables. Boldface *p*-values are significant at α = 0.05

Table 2 showed the baseline metabolic syndrome characteristics. In total, 46.6% of residents with depressive symptoms and 38.6% without depressive symptoms had metabolic syndrome (*P* < 0.001). More patients with subjective depressive symptoms met the HDL criteria, TG and Waist circumference for metabolic syndrome. We performed further analysis by genders and found that in women depressive symptoms were significantly associated with metabolic syndrome (51.9% vs. 45.0%, *P* < 0.001) and each of the components of metabolic syndrome like high TG and waist circumference, increased glucose. However, there was no significant association between MetS and depressive symptoms and metabolic disorders in men. In terms of the continuous components of metabolic syndrome, all factors, with the exception of HDL-C and waist circumference, were associated with depressive symptoms in women. However, there was no such relationship existed in men.

**Table 2:** Metabolic syndrome characteristics of the study population according to sex and depressive symptoms

	Total		*P*-value^a^	Men		*P*-value^a^	Women		*P*-value^a^
	Depressed	Not depressed		Depressed	Not depressed		Depressed	Not depressed	
	*N* = 685	*N* = 10745		*N* = 186	*N* = 5093		*N* = 499	*N* = 5652	
Metabolic syndrome^b^, *n* (%)	319 (46.6)	4143 (38.6)	<0.001	60 (32.3)	1598 (31.4)	0.427	259 (51.9)	2545 (45.0)	0.002
Waist circumference component^c^, *n* (%)	347 (50.7)	4552 (42.4)	<0.001	51 (27.4)	1410 (27.7)	0.506	296 (59.3)	3142 (55.6)	0.059
TG component^d^, *n* (%)	260 (38.0)	3412 (31.8)	<0.001	65 (34.9)	1617 (31.7)	0.200	195 (39.1)	1795 (31.8)	0.001
HDL component, *n* (%)	249 (36.4)	3132 (29.1)	<0.001	35 (18.8)	820 (16.1)	0.186	214 (42.9)	2312 (40.9)	0.208
Hypertension component, *n* (%)	469 (68.5)	7204 (67.0)	0.234	137 (73.7)	3645 (71.6)	0.298	332 (66.5)	3559 (63.0)	0.062
Glucose component, *n* (%)	331 (48.3)	5048 (47.0)	0.26	91 (48.9)	2575 (50.6)	0.358	240 (48.1)	2473 (43.8)	0.034
Number of components, *n*	2.42 ± 1.43	2.17 ± 1.34	<0.001	2.04 ± 1.33	1.98 ± 1.26	0.516	2.56 ± 1.44	2.35 ± 1.40	0.001
Waist circumference, mean ± SD, cm	82.37 ± 9.92	82.45 ± 9.83	0.833	83.60 ± 10.00	83.80 ± 9.76	0.778	81.91 ± 9.86	81.23 ± 9.72	0.136
TG, mean ± SD, mg/dl	1.75 ± 1.51	1.63 ± 1.49	0.038	1.61 ± 1.10	1.66 ± 1.68	0.672	1.80 ± 1.64	1.60 ± 1.30	0.001
HDL^e^, mean ± SD, mg/dl	1.40 ± 0.40	1.41 ± 0.38	0.668	1.40 ± 0.47	1.41 ± 0.42	0.742	1.40 ± 0.37	1.41 ± 0.34	0.716
^f^Systolic blood pressure, mean ± SD, mmHg	143.74 ± 25.49	141.61 ± 23.32	0.021	145.94 ± 24.52	143.55 ± 22.56	0.158	142.92 ± 25.82	139.87 ± 23.86	0.006
^f^Diastolic blood pressure, mean ± SD, mmHg	82.29 ± 11.86	82.03 ± 11.76	0.569	83.79 ± 11.91	83.76 ± 11.81	0.977	81.74 ± 11.80	80.47 ± 11.50	0.019
^g^Fasting glucose, mean ± SD, mg/dl	6.08 ± 1.75	5.89 ± 1.62	0.004	5.99 ± 1.59	5.95 ± 1.67	0.679	6.10 ± 1.81	5.84 ± 1.58	<0.001

Abbreviations: *SD* standard deviation; *TG* triglyceride; *HDL* high-density lipoprotein cholesterol

^a^Two-tailed Student’s *t*-test for normally distributed variables, Mann–Whitney *U*-test for skewed variables, and chi-square test for categorical variables. Boldface *p*-values are significant at α = 0.05

^b^the definition of MetS was according to previous study [13]

^c^Waist circumference threshold for abdominal obesity for Asian population, ≥90 cm for men and ≥80 cm for women

^d^Serum TG ≥150 mg/dL or antilipidemic medication

^e^Serum HDL <40 mg/dL for men and <50 mg/dL for women

^f^Systolic blood pressure ≥130 mm Hg or diastolic blood pressure ≥ diastolic blood pressure ≥85 mm Hg or antihypertensive medication

^g^Fasting plasma glucose ≥100 mg/dL or antidiabetic medication

### Association between MetS and depressive symptoms and each of its components

**Fig. 1:**
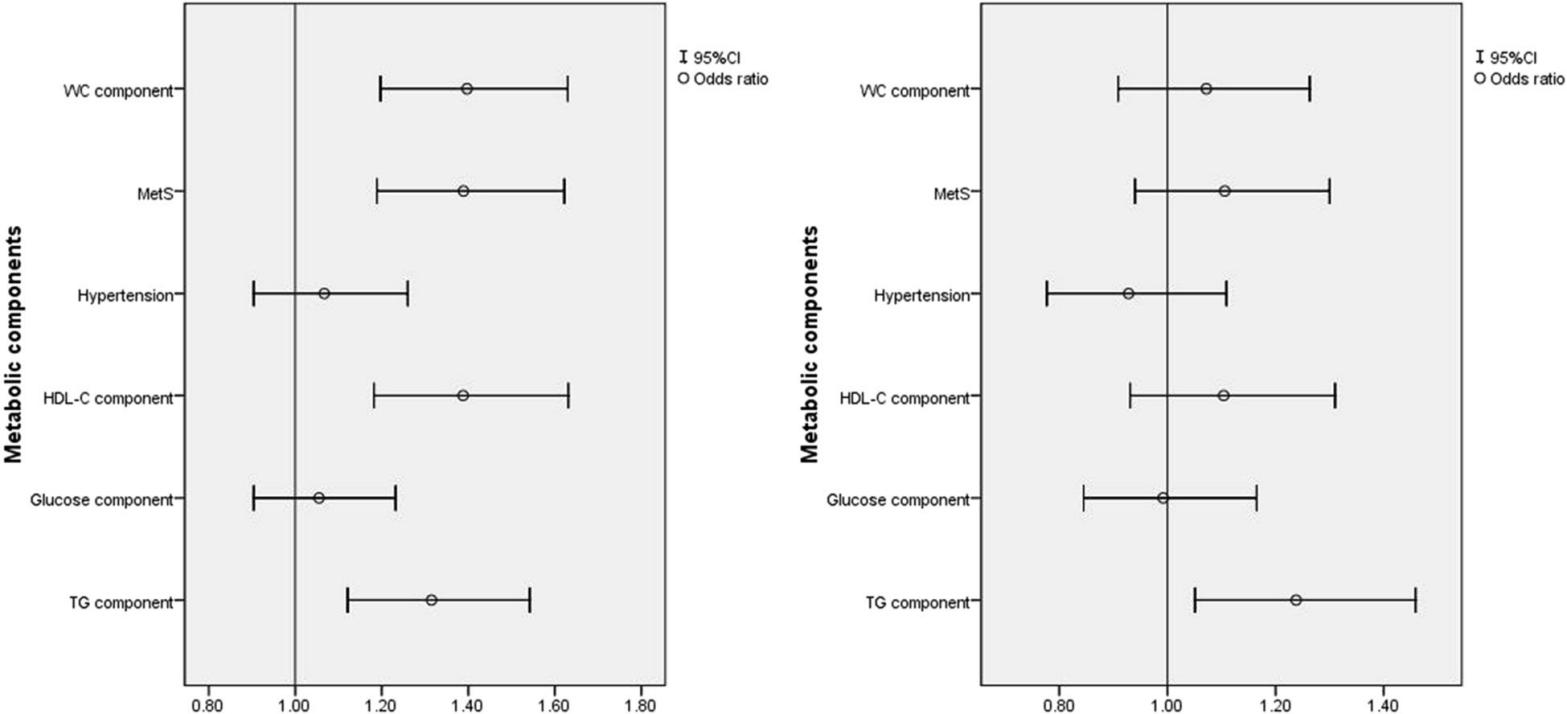
Relationship between depressive symptoms and metabolic components with and without multivariate adjust in general population. Adjusted for age, sex, ethnicity, education level, family income, current smoking and drinking status, physical activity

**Fig. 2:**
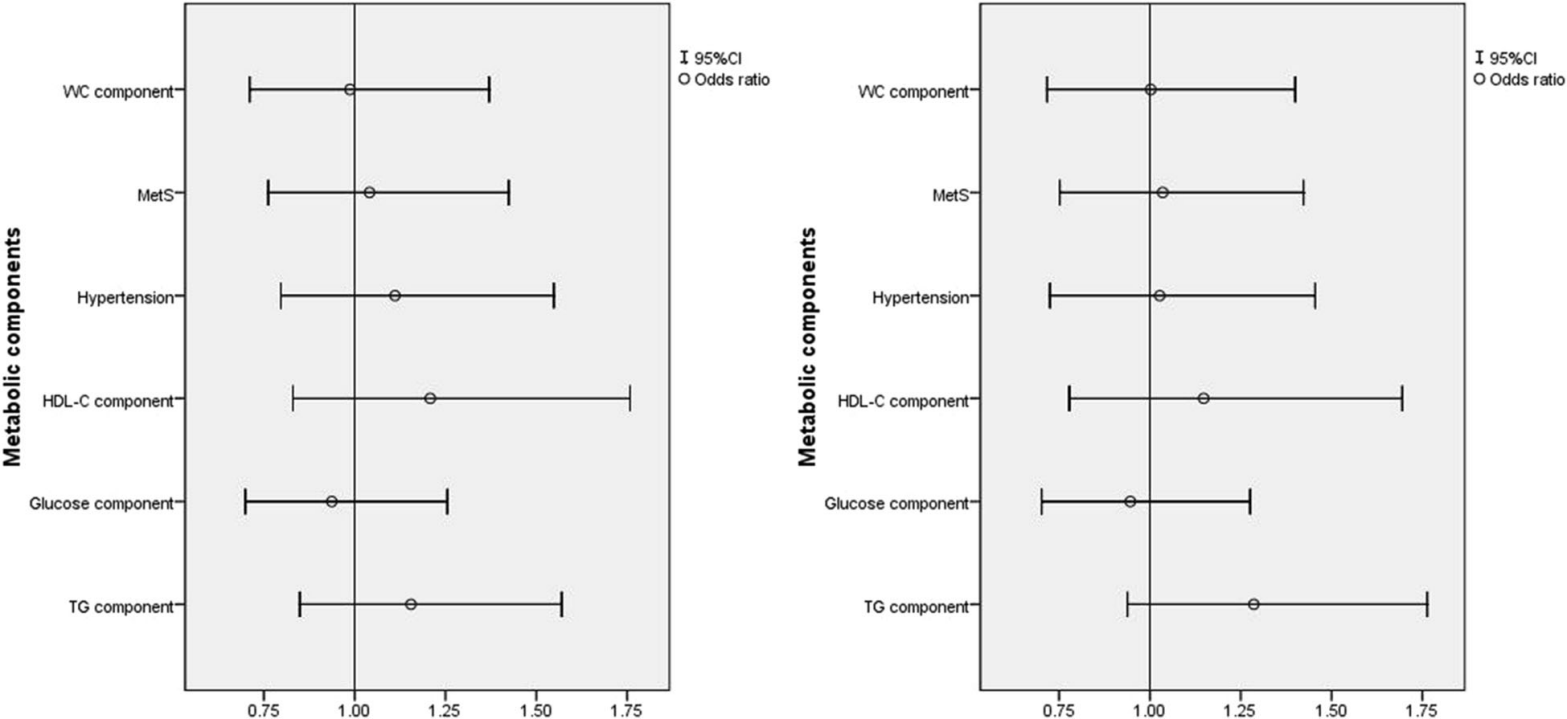
Relationship between depressive symptoms and metabolic components with and without multivariate adjust in men. Adjusted for age, sex, ethnicity, education level, family income, current smoking and drinking status, physical activity

**Fig. 3:**
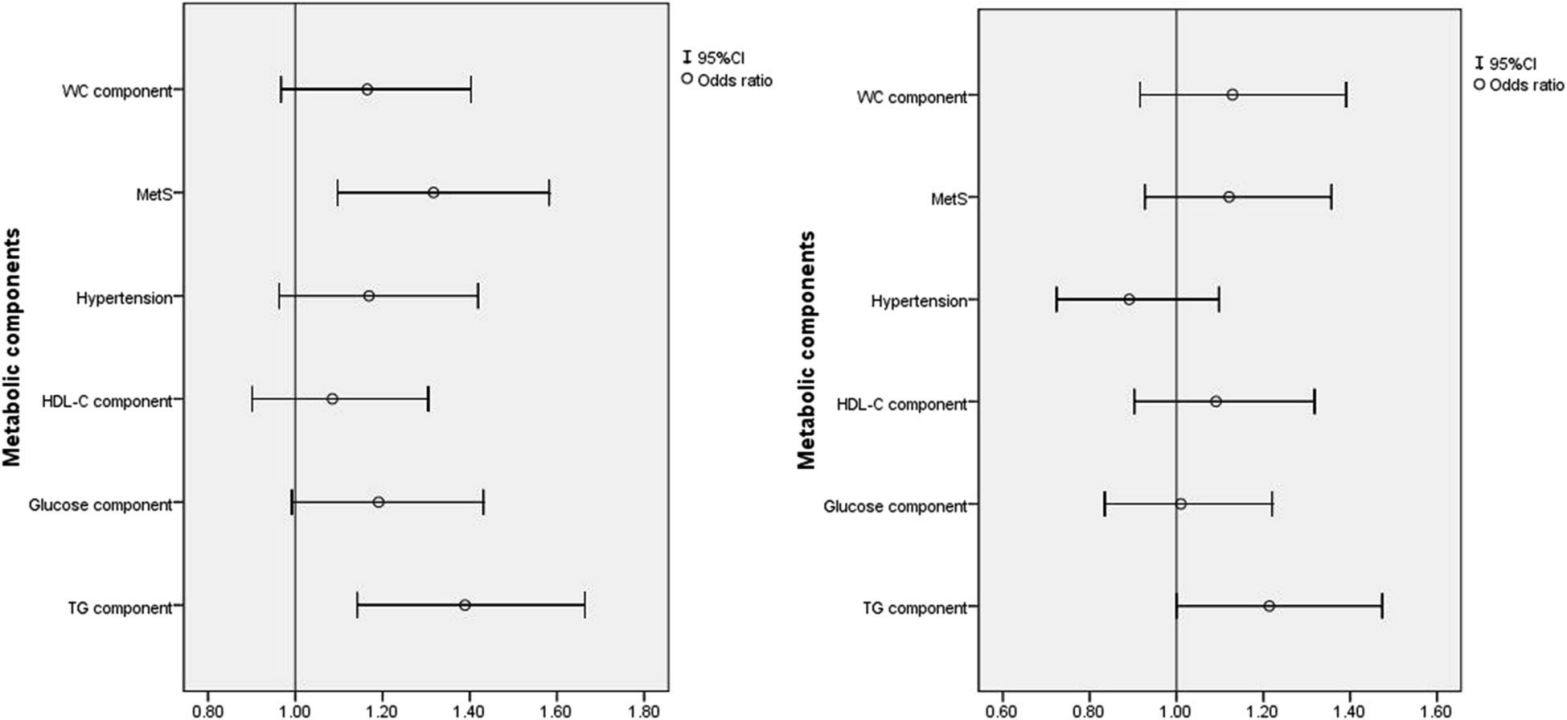
Relationship between depressive symptoms and metabolic components with and without multivariate adjust in women. Adjusted for age, sex, ethnicity, education level, family income, current smoking and drinking status, physical activity

In the total study population, depressive symptoms were positively associated with metabolic syndrome and other metabolic components. After adjusting for confounding factors including sex, only the relationship with TG component was significant but not with MetS (Fig. 1). Stratification by sex showed different patterns of associations (Figs. 2 and 3) In women, depressive symptoms were associated with TG component only, but in men, there was no association between depressive symptoms and TG component. After adjusting for age, marriage, cigarette smoking, alcohol use, exercise and education, depressive symptoms were still associated with TG component (OR = 1.214, 95%CI = 1.000, 1.474, *p* = 0.05).

## Discussion

The results of this study revealed that the prevalence of metabolic syndrome among depressed residents was much higher than those without depressive symptoms (for general 44.6% vs. 38.6%, *P* < 0.001; for men 32.3% vs. 31.4%, *P* = 0.427; for women 51.9% vs. 45.0%, *P* = 0.002). However, depressive symptoms were associated with TG component in general population especially in women after adjusted for confounding factors. Residents with depressive symptoms had higher risk of metabolic syndrome than those without depressive symptoms. But once adjusted for possible confounders, the association did not reach statistical difference. Focusing on the individual components, the TG component was associated with depressive symptoms after adjusted for varies confounding factors in women. However, after adjusted for menopausal status, relationship between depressive symptoms and TG component was no longer statically existed.

The prevalence of depressive symptoms in general population of China had been reported elsewhere by using different self-completed questionnaires. A most recent meta-analysis showed that the lifetime prevalence of major depressive disorder (MDD) was 3.3% (95%CI: 2.4–4.1), the 12-month prevalence was 2.3% (95%CI: 1.8–3.4) and the current prevalence was 1.6% (95%CI: 1.2–1.9) in China [18]. The prevalence of MDD between different regions in China varied from 1% to 6.5% [19–21]. MDD prevalence in urban China was 6.0% with a relatively low diagnosed rate (8.3%) and only 51.5% currently used prescription medication for depression [22]. Our study reported that the prevalence of depressive symptom was 5.9% in rural Northeast China which seemed to be close to the urban areas [23]. Many previous studies also found that the current prevalence of MDD was significantly higher in rural areas (2.0%, 95%CI: 1.2–2.9) than in urban areas (1.7%, 95%CI: 0.8–2.7) [18]. Zhou X and colleagues explained that rural residents were more likely to suffer poor health status, have chronic disease, and to be poor which was strongly associated with depression [23].

Similar with the variety of MDD prevalence, there was a wide range of MetS prevalence from different regions. A relatively higher prevalence of MetS has been noted in several coastal and inland areas, including Sichuan (23.8%), Shanghai (29.34%), Beijing (23.2%) and Guangdong (26.7%) [24–26]. Nevertheless, our study found that the prevalence of MetS (39.0%) in rural Northeast China was even higher than urban areas. Furthermore, the prevalence of MetS among depressed residents (46.6%) was significantly higher than those without depressive symptoms (38.6%) and the general population (39.0%). Even though in the present study, we did not confirmed that depressed residents had significantly higher risk of MetS like many previous studies.

Those studies that claimed depressed residents had higher risk of MetS reported that the disturbances in the hypothalamic-pituitary- adrenal (HPA) axis, chronic inflammation, the excitement of sympathetic nervous system and unhealthy living habit might partially explain it [27, 28]. However, Douglas KM and colleagues investigated that depressive symptoms were only weakly correlated with CRP. Furthermore, after adjusting for BMI, there was no significant relationship between CRP and depression. They assumed that the relationship between depressive symptoms and cardiac diseases might be mediated by BMI but not CRP levels [29]. Interestingly, in our study, when we used ATP-III definition to define MetS, there was positive relationship between depressive symptoms and MetS (data not shown). And the differences between ATP-III criteria and the criteria we used in the present study was the definition of abdominal obesity and elevated fasting glucose. Hence, it suggested that except for endocrine regulation, obesity including general obesity and abdominal obesity might partially related with the association between depressive symptoms and MetS. However, in the present study, we did not evaluate the associated endocrine index. So we cannot explain why MetS was not significantly associated with depressive symptoms in our study. Further study is required to figure out the possible explanations.

As far as we know, there have been no reports from China investigating the association of MetS and depressive symptoms. However, there are studies from other Asian countries [30, 31]. In the same way, among rural residents, our findings were in agreement with researches showing that depressive symptoms were more prevalent among residents with metabolic syndrome [32, 33]. However, after possible confounders were taken into consideration, this association was no longer existed which was not in line with other previous studies [34–36]. Similarly, unlike other previous studies, our study found a positive association between depressive symptoms and TG component in general population after adjusting for potential confounders. However, after stratified by gender, depressive symptoms were associated with TG component only in women but not in men. Yasumi Kimura and colleagues claimed that depressive symptoms was associated with fasting hyperglycemia while Sang Jin Rhee announced that HDL component was associated with depressive symptoms in women [37, 38]. In our study, women with depressive symptoms had significantly higher value of glucose, TG and HDL components than those without them which was similar with the previous studies [37, 38]. In especial, the prevalence of elevated TG in our study was dramatically higher than the other studies also held in Asia areas. This might partially explain the inconsistence of our finding.

There are several limitations to the present study. First, due to the cross-sectional design of our study, we cannot make inferences about causality. Depressive symptoms may be a cause or a consequence of metabolic alterations. Second, this study used a questionnaire and did not use a comprehensive psychiatric evaluation. We did not confirm a definitive diagnosis of depression.

## Conclusions

In conclusion, our study estimated the prevalence of MetS, its components and depressive symptoms in general residents from rural Northeast China and revealed that a higher prevalence of MetS among depressive ones. After adjusted for possible confounders, data showed that depressive symptoms were associated with TG component only. This emphasizes, especially in women, the importance of screening for and diagnosing depression and routine lipid assessment in those depressive women.

## Abbreviations

CIs: Confidence intervals
FPG: Fasting plasma glucose
HDL-C: High-density lipoprotein cholesterol
LDL-C: Low-density lipoprotein cholesterol
ORs: Odds ratios
PDH: Previously diagnosed hypertension
PHQ-9: Patient Health Questionnaire-9
PUH: Previously undiagnosed hypertension
TC: Total cholesterol
TG: Triglyceride
